# Supplementary material for: Alkaline Stress Induces Different Physiological, Hormonal and Gene Expression Responses in Diploid and Autotetraploid Rice
Source: Int J Mol Sci. 2022 May 16;23(10):5561. doi: 10.3390/ijms23105561 (PMC9142035; doi:10.3390/ijms23105561)
Supplement: Supplementary file 1 [file ijms-23-05561-s001.zip › Supplementary Materials_legends.pdf]

Supplementary Materials:

Figure S1: The morphological variation between 9311-2x and 9311-4x. (a) Chromosome counts of 9311-2x and 9311-4x. Root-tip cells were used for karyotype analysis. Scale bar = 1 μM. (b) Phenotypic variation between 9311-2x and 9311-4x during filling stage. Scale bar = 10 cm. (c) Phenotypic variation of seeds between 9311-2x and 9311-4x. Scale bar = 1cm. (d) The detection of plant height, dry weight and fresh weight in 93-11-2x and 93-11-4x after alkaline stress. A single asterisk (*) indicates that the difference was significant ($P < 0.05$) and ** indicates that the difference was extremely significant ($P < 0.01$)

Figure S2: The expression of 10 genes was amplified by real time quantitative polymerase chain reaction (qRT-PCR) for the verification of the transcriptome results.

Figure S3: (a) Gene Ontology (GO) classification and enrichment analysis of differentially expressed genes in root between 9311-2x and 9311-4x. (b) Kyoto encyclopedia of genes and genomes (KEGG) pathway with significantly enriched differentially expressed genes (DEGs) in shoot between 9311-2x and 9311-4x. (c) The expression of genes related to the peroxidase superfamily protein in rootd between 9311-2x and 9311-4x. The heatmap with yellow color was the genes expression level (read counts per million with log2 value), whereas with white color was fold change (log2 value) of DEGs, respectively. * indicates the difference was significant ($P < 0.05$).

Figure S4: The expression of genes related to phytohormones of root between 9311-2x and 9311-4x in mock and high pH conditions. The genes were released by Mapman software with DEGs mapping. The heatmap with yellow color was the genes expression level (read_counts per million with log2 value), whereas with white color was fold change (log2 value) of DEGs, respectively. * indicates the difference was significant ($P < 0.05$).

Figure S5: The expression of ionic transport-related genes of root and shoot between 9311-2x and 9311-4x in mock and high pH conditions.

Figure S6: The expression of transcription factors related genes of root and shoot between 9311-2x and 9311-4x in mock and high pH conditions.

Table S1: RNA-seq Data quality control.

Table S2. The list of qRT-PCR primer sequences.

Table S3. The list of DEGs in GO terms.

Table S4. The list of DEGs in KEGG term.

Table S5. The list of DEGs with phytohormone-related genes.

Table S6. The list of DEGs with peroxidase superfamily protein and ionic transport-related genes.

Table S7. The list of DEGs with transcription factors-related genes.
