# Supplementary figures and images for: Alkaline Stress Induces Different Physiological, Hormonal and Gene Expression Responses in Diploid and Autotetraploid Rice
Source: Int J Mol Sci. 2022 May 16;23(10):5561. doi: 10.3390/ijms23105561 (PMC9142035; doi:10.3390/ijms23105561)

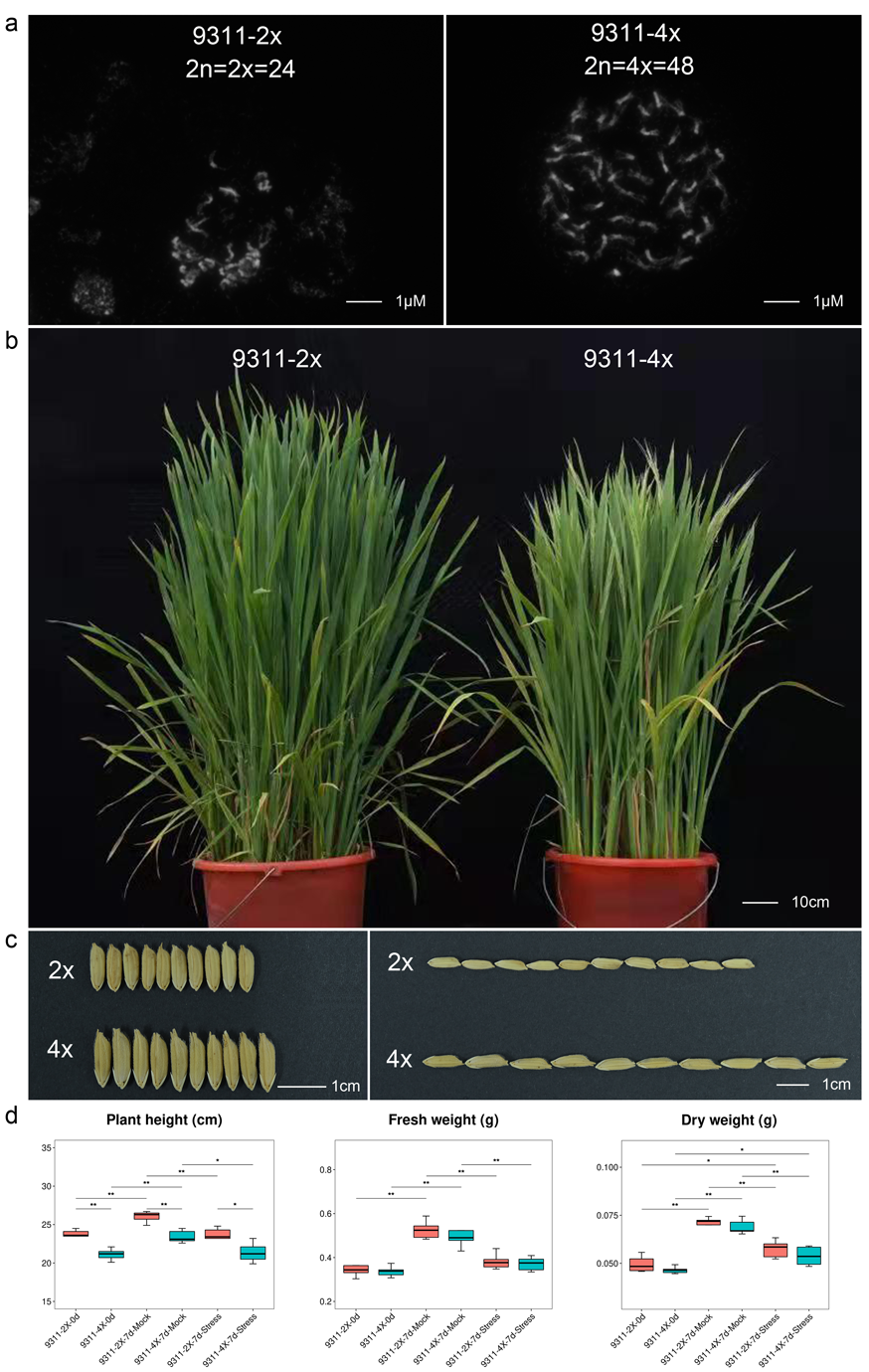

Supplement: Supplementary file 1 [file ijms-23-05561-s001.zip › Supplementary_FigureS1.tif]

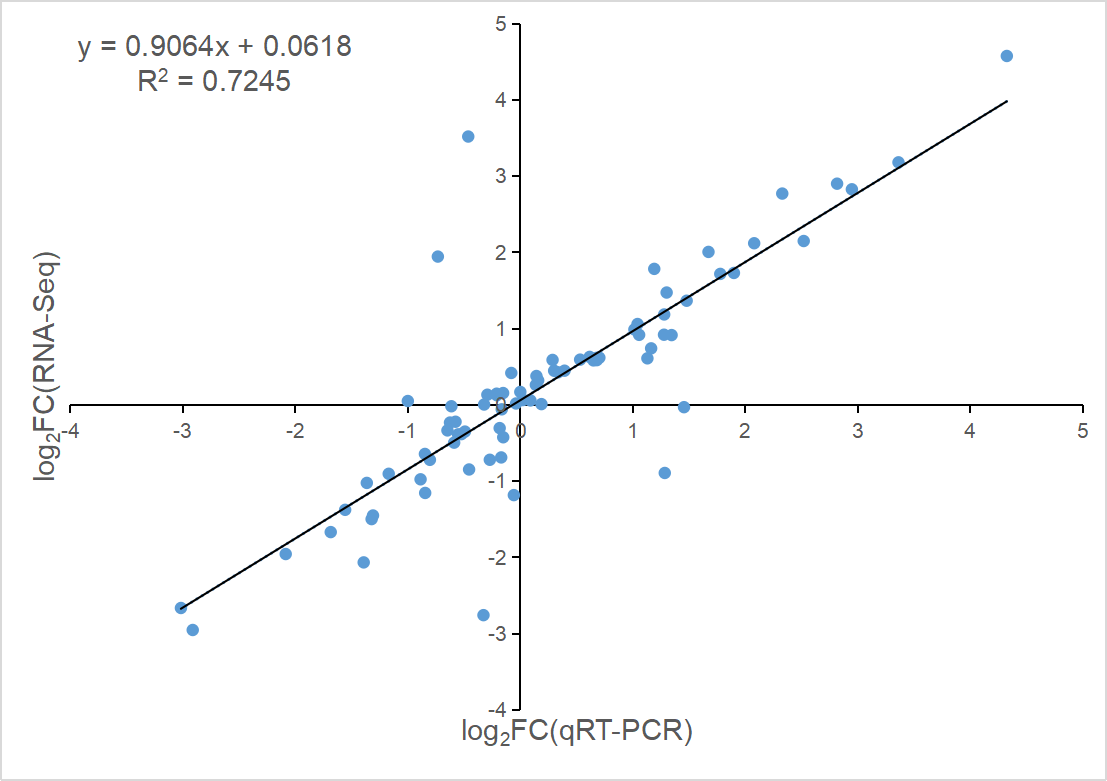

Supplement: Supplementary file 1 [file ijms-23-05561-s001.zip › Supplementary_FigureS2.tif]

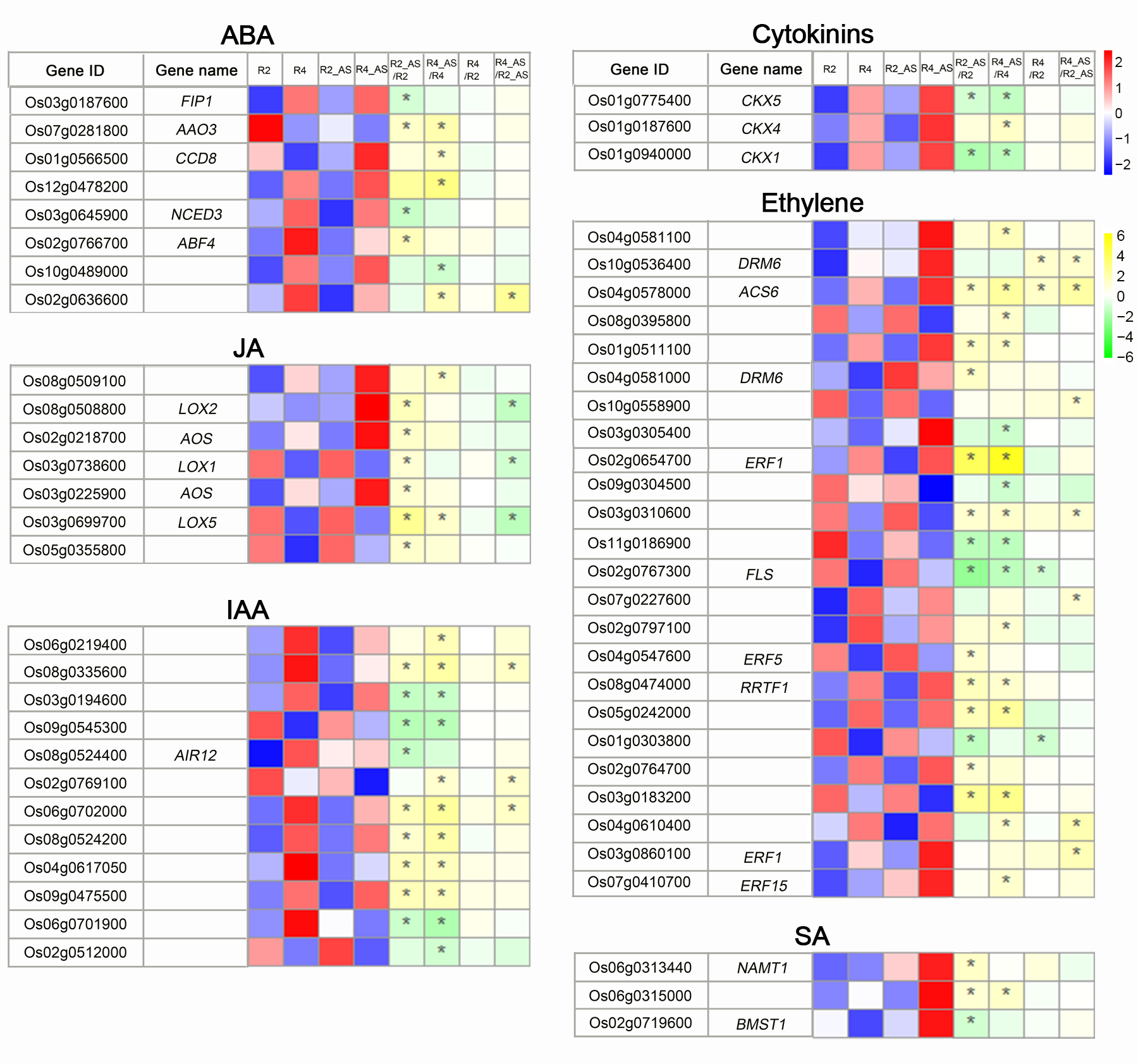

Supplement: Supplementary file 1 [file ijms-23-05561-s001.zip › Supplementary_FigureS4.tif]

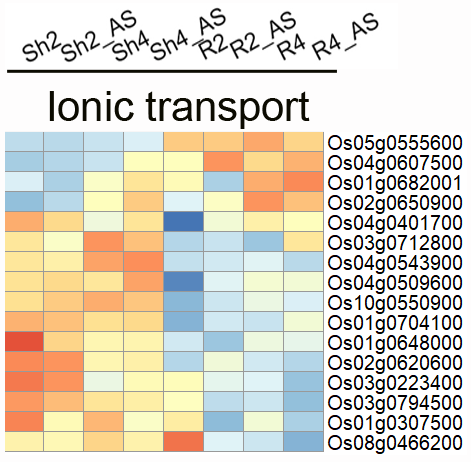

Supplement: Supplementary file 1 [file ijms-23-05561-s001.zip › Supplementary_FigureS5.tif]

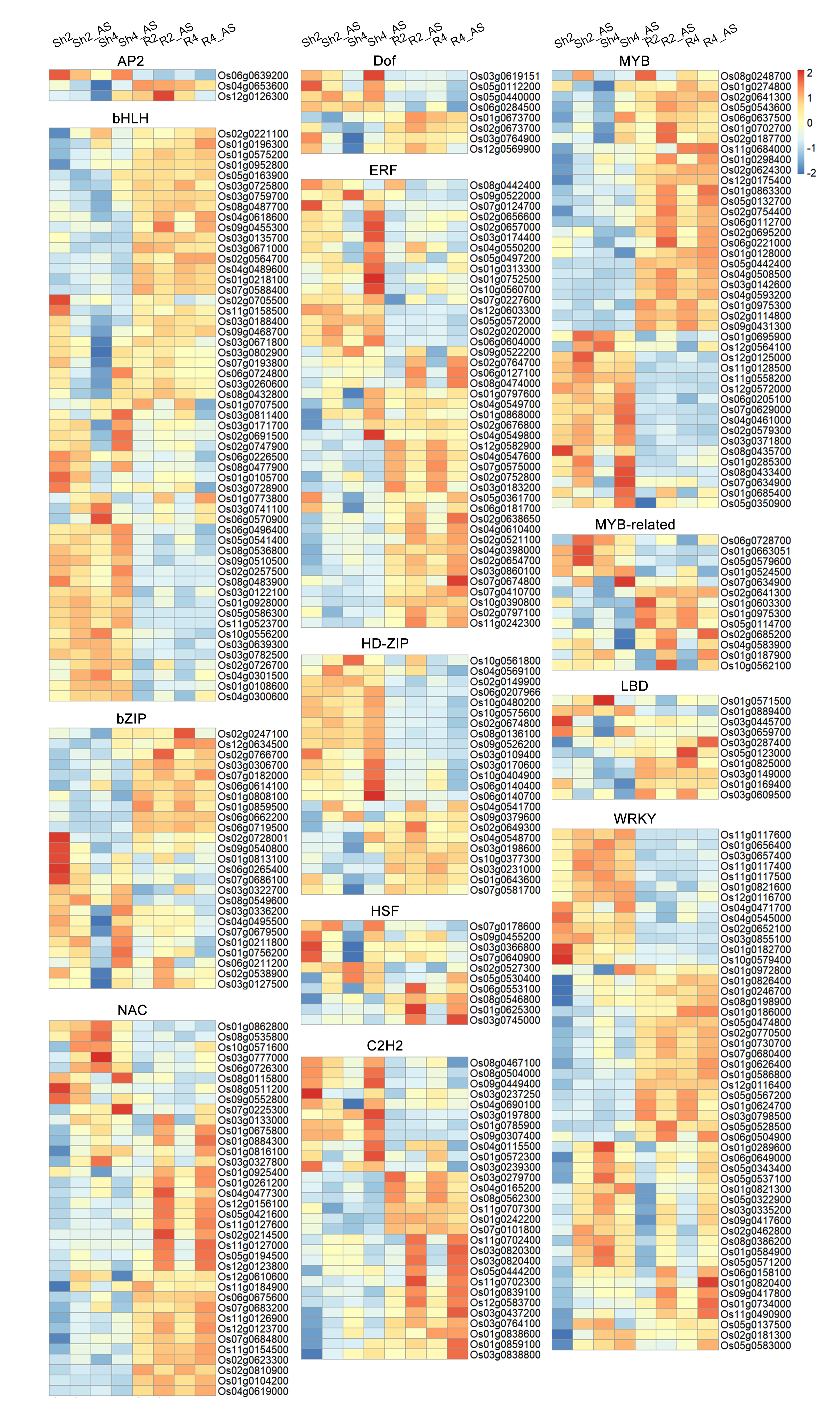

Supplement: Supplementary file 1 [file ijms-23-05561-s001.zip › Supplementary_FigureS6.tif]
